# Flow-Induced Transcriptomic Remodeling of Endothelial Cells Derived From Human Induced Pluripotent Stem Cells

**DOI:** 10.3389/fphys.2020.591450

**Published:** 2020-10-15

**Authors:** Emmi Helle, Minna Ampuja, Laura Antola, Riikka Kivelä

**Affiliations:** ^1^Stem Cells and Metabolism Research Program, Faculty of Medicine, University of Helsinki, Helsinki, Finland; ^2^New Children’s Hospital, and Pediatric Research Center Helsinki University Hospital, Helsinki, Finland; ^3^Wihuri Research Institute, Helsinki, Finland

**Keywords:** endothelial cells, induced pluripotent stem cells, shear stress, flow, single-cell RNA sequencing, RNA sequencing

## Abstract

The vascular system is essential for the development and function of all organs and tissues in our body. The molecular signature and phenotype of endothelial cells (EC) are greatly affected by blood flow-induced shear stress, which is a vital component of vascular development and homeostasis. Recent advances in differentiation of ECs from human induced pluripotent stem cells (hiPSC) have enabled development of *in vitro* experimental models of the vasculature containing cells from healthy individuals or from patients harboring genetic variants or diseases of interest. Here we have used hiPSC-derived ECs and bulk- and single-cell RNA sequencing to study the effect of flow on the transcriptomic landscape of hiPSC-ECs and their heterogeneity. We demonstrate that hiPS-ECs are plastic and they adapt to flow by expressing known flow-induced genes. Single-cell RNA sequencing showed that flow induced a more homogenous and homeostatically more stable EC population compared to static cultures, as genes related to cell polarization, barrier formation and glucose and fatty acid transport were induced. The hiPS-ECs increased both arterial and venous markers when exposed to flow. Interestingly, while in general there was a greater increase in the venous markers, one cluster with more arterial-like hiPS-ECs was detected. Single-cell RNA sequencing revealed that not all hiPS-ECs are similar even after sorting, but exposing them to flow increases their homogeneity. Since hiPS-ECs resemble immature ECs and demonstrate high plasticity in response to flow, they provide an excellent model to study vascular development.

## Introduction

Endothelial cells (ECs) are present in all tissues, and they regulate the development, growth and function of all organs. Endothelial dysfunction has been implicated as a major cause of many developmental defects and several adulthood diseases (Feng et al., 2002). Human induced pluripotent stem cell-derived endothelial cells (hiPS-EC) show great promise for disease modeling, drug discovery and regenerative medicine, as they can be obtained from healthy individuals as well as from patients harbouring genetic variants or diseases of interest. A number of protocols have been described to derive ECs from hiPSCs. When compared to primary ECs, hiPS-ECs have more embryonic-like gene signatures, but they demonstrate similar functional properties as primary ECs, such as 3D tube formation, barrier function and response to inflammatory stimuli (Orlova et al., 2014a,b; Halaidych et al., 2018).

ECs are constantly exposed to shear stress *in vivo.* Shear stress regulates diverse physiological processes in health and disease. Laminar shear stress induced by blood flow is an essential regulator of blood vessel development (Campinho et al., 2020), and it promotes endothelial cell quiescence, which is required for vascular homeostasis (Baeyens et al., 2016). Multiple pathways classically known to be involved in embryonic development, such as BMP–TGFβ, WNT, NOTCH, HIF1α, TWIST1, and HOX family genes, are regulated by shear stress in adult arteries. Mechanical activation of these pathways likely evolved to orchestrate vascular development, but they can also drive atherosclerosis upon disturbed flow and low shear stress.

Even though hiPSC-derived ECs do not fully recapitulate the phenotype and function of adult ECs, they provide an excellent tool to model tissue development *in vitro*. While several studies have demonstrated the transcriptomic effects of shear stress in primary ECs, data on effects of flow on hiPS-EC are still scarce. In this study, we examined how hiPS-ECs respond and adapt to flow, and used single-cell RNA sequencing (scRNASeq) to evaluate the heterogeneity of response. This is important as hiPS-ECs are increasingly studied for both *in vitro* modeling as well as for transplantation to patients with vascular diseases.

## Materials and Methods

### Data Availability

The RNA sequencing datasets generated for this study are deposited in the Gene Expression Omnibus (GEO) database with accession numbers GSE150741 and GSE150740.

### hiPS Cell Lines

Three healthy human induced pluripotent stem cell lines (HEL47.2, HEL46.11, and HEL24.3) were obtained from the Biomedicum Stem Cell Center. The cell lines were created by using retroviral/Sendai virus transduction of Oct3/4, Sox2, Klf4, and c-Myc, as described previously (Trokovic et al., 2015a,b; Saarimäki-Vire et al., 2017). In addition, the hiPSC line K1 was a kind gift from Prof. Anu Wartiovaara group.

### hiPSC Culture

hiPSCs were maintained in Essential 8 media (A1517001, Thermo Fisher Scientific) on thin-coated Matrigel (354277, dilution 1:200; Corning, Corning, NY, United States). The cells were passaged using EDTA.

### hiPS-EC Differentiation

Endothelial cell differentiation was conducted based on the protocol by Giacomelli et al. (2017) with slight modifications. The BPEL medium ingredients were purchased from the same vendors as mentioned in the article, except for BSA (A7030, Sigma) and PVA (362607, Sigma). Briefly, 125,000 – 175,000 cells/well in a 6-well plate were plated on day 0. On day 1, the medium was changed to BPEL with 20 ng/ml BMP4 (120-05ET, Peprotech), 20 ng/ml Activin A (AF-120-14E–50 μg, Peprotech) and 4 μmol/L CHIR (S2924, Selleckhem). On day 3, the medium was changed to BPEL with 50 ng/ml VEGF (produced in-house) and 5 μmol/L IWR-1 (I0161, Sigma). On day 6, medium was changed to BPEL with 50 ng/ml VEGF and the cells were maintained in this medium until they were sorted. 50 ng/ml VEGF was maintained in all hiPS-ECs cultures unless otherwise indicated.

### hiPS-EC Sorting

After differentiation, hiPS-ECs were sorted using magnetic beads with an antibody against CD31 (130-091-935, Miltenyi Biotec), according to the manufacturer’s protocol. The concentration of the cells was counted with Bio-Rad TC10 or TC20 Automated Cell Counter. The cells were immediately used for experiments.

### hiPS-EC Exposure to Flow

After sorting, 2.5–3.5 × 10^5 hiPS-ECs were plated on an Ibidi μ-Slide I Luer (80176, Ibidi). 4.0–6.0 × 10^5 hiPS-ECs were plated in one well in 6-well plate (static control). After 24 h, the cells on Ibidi slide were subjected to laminar shear stress of 15 dyn/cm^2^ by using the Ibidi Pump System (10902, Ibidi). After 24 h of exposure to flow, the cells were processed either for bulk RNA-sequencing or single-cell RNA-sequencing. The static control cells were processed at the same time. For bulk RNA-sequencing, the cells were collected into the RA1 lysis buffer and extracted using the Nucleospin RNA Plus Extraction kit (740984, Macherey-Nagel). Each experiment (whether for scRNASeq or bulk RNASeq) consists of one differentiation round from each hiPS cell line.

### Immunofluorescence Staining

Cells were fixed with 4% PFA and stained with VE-cadherin (2500, Cell Signaling Technology). Nuclei were visualized with DAPI or Hoechst. Stained cells were imaged with fluorescent or confocal microscopes (Zeiss AxioImager and Zeiss LSM 780).

### Matrigel Tube Assay

48-well plate was coated with 100 μl of Matrigel per well. After gelling of Matrigel, 90,000 hiPS-ECs/well (HEL24.3 and HEL47.2) or 30,000 HUVECs/well were added on top of the Matrigel-covered wells. The cells were allowed to attach and grow. Phase-contrast images were taken at 24, 48, and 72 h.

### LDL Uptake

Atto-labeled LDL oxidized with 10 μmol/L CuSO4 (20 h, 37C°) (courtesy of Dr. Katariina Öörni lab) was applied to hiPS-ECs on coverslips in a 24-well plate for 20 h (7.5 μg/well). The coverslips were fixed and imaged with a Zeiss LSM 780 confocal microscope.

### Processing of Cells for Single-Cell RNA-Sequencing

The cells were detached using Accutase (A6964, Sigma) and concentration of the cell suspension was measured. The cells were washed once with PBS containing 0.04% BSA, and then resuspended in PBS with 0.04% BSA to a final concentration of 0.79–1.0 × 10^6 cells/ml. The cells were passed through a 35 μm strainer (352235, Corning) and kept on ice until processing for the 10X Genomics Single Cell Protocol at the Institute of Molecular Medicine Finland (FIMM). At FIMM, the concentration and viability of cells was calculated one more time with Luna Automated Cell Counter and 4000 cells/sample were processed.

### Single-Cell RNA-Sequencing

Single-cell gene expression profiles were studied using the 10× Genomics Chromium Single Cell 3′RNAseq platform. The Chromium Single Cell 3′RNAseq run and library preparation were done using the Chromium Single Cell 3′ Reagent version 2 chemistry. The sample libraries were sequenced on Illumina NovaSeq 6000 system. 4000 cells and 50,000 PE/cell were analyzed.

Data processing and analysis were performed using 10× Genomics Cell Ranger v2.1.1 pipelines. The “cellranger mkfastq” pipeline was used to produce FASTQ (raw data) files. The “cellranger count” was used to perform alignment, filtering and UMI counting. mkfastq was run using the Illumina bcl2fastq v2.2.0 and alignment was done against human genome GRCh38. Cellranger aggr pipeline was used to combine data from multiple samples into an experiment-wide gene-barcode matrix and analysis.

Analyses were performed with Seurat R package version 3.0.1 (Butler et al., 2018). Cells, in which more than 1000 genes were detected, were included. Seurat function CellCycleScoring was used to assign cell cycle scores (iG2/M scores and S scores), which then were used to regress out cell cycle effect. Normalization and variance stabilization was done with SCTransform in Seurat (vars.to.regress was used to remove confounding sources of variation including mitochondrial mapping percentage, and cell cycle scores) (Hafemeister and Satija, 2019). Principal component analysis (PCA) was performed on the highly variable genes. The first 30 PCs were used for uniform manifold approximation (UMAP). Differential expression for each subpopulation in the scRNASeq data was performed using the FindAllMarkers function (Wilcoxon Rank Sum test) in Seurat, and FindMarkers was used to distinguish different conditions. Cells were clustered based on their expression profile setting the resolution to 0.5, which led to 11 clusters which were quite clearly distinguished in the UMAP. Established markers on The Human Protein Atlas and published literature were used to annotate cell types.

### Single-Cell RNA-Sequencing – Data Presentation

For clarity, we present the scRNASeq results in the main figures for the cell line HEL47.2. Only results, which were statistically significant in both cell lines, are presented, unless otherwise stated. The corresponding results for the cell line HEL24.3 are presented in the Supplementary Material.

### Bulk RNA-Sequencing

RNA samples were sequenced at Biomedicum Functional Genomics Unit (FuGU) with Illumina NextSeq sequencer (Illumina, San Diego, CA, United States) in High output run using NEBNext^®^ Ultra^TM^ II Directional RNA Library Prep Kit for Illumina. The sequencing was performed as single-end sequencing for read length 75 bp. The count data was used to calculate differential expression statistics with the DESeq2 software in the R environment. Genes with an adjusted *p*-value for the log2-fold change <0.05 were considered significant. Gene Ontology (GO) analysis was performed using DAVID Bioinformatics Resources 6.8.

## Results

### hiPS-EC Characterization

**FIGURE 1:**
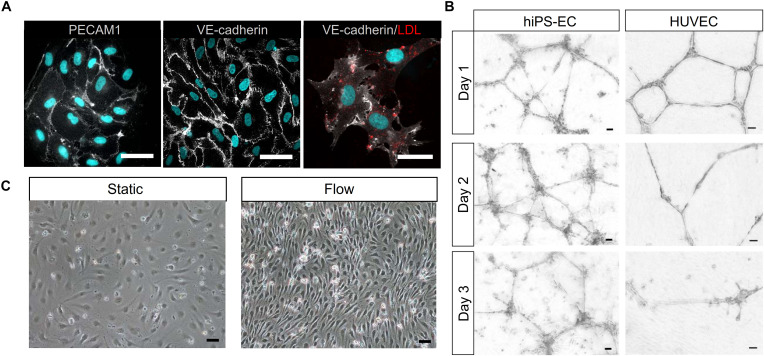
Characterization of hiPS-ECs. **(A)**, PECAM1 and VE-cadherin IF staining of hiPS-ECs (white) and uptake of oxLDL by hiPS-ECs (red). Nuclei are shown in blue. Scale bar 50 μm. **(B)**, Matrigel tube assay of hiPS-ECs and HUVECs (scale bar 50 μm). The cells were plated on Matrigel and imaged every day for 72 h. **(C)**, Phase-contrast images of hiPS-ECs after 24 h exposure to flow-induced shear stress and under static conditions. The cells in both conditions are from the same differentiation batch and images have been taken at the same time point just before processing them for scRNASeq. Scale bar 50 μm.

Immunofluorescence staining of the hiPS-ECs showed strong expression of VE-cadherin and PECAM1 in cell-cell junctions (Figure 1A). In addition, the hiPS-ECs were able to take up oxidized LDL (Figure 1A), demonstrating their functionality. Matrigel tube assay showed that the hiPS-ECs formed tubes in 3D Matrigel assay similarly to HUVECs (Figure 1B). Interestingly, although the hiPS-EC tubes were not as uniform, they lasted longer than the HUVEC-derived structures. In 2D culture, the hiPS-ECs had a cobblestone-like morphology similar to primary ECs, and upon exposure to shear stress they aligned in response to flow (Figure 1C).

### Single Cell Profiling of hiPS-ECs Grown in Static Conditions and Under Flow

To analyze the heterogeneity and identity of the hiPS-ECs, we performed scRNASeq in two independent hiPS cell lines from healthy donors (HEL47.2 and HEL24.3). The results from HEL47.2 are presented in the main figures and the results from HEL24.3 in the Supplementary Material. In general, the results were highly similar in both cell lines and only genes and pathways found to be affected in both lines are reported.

**FIGURE 2:**
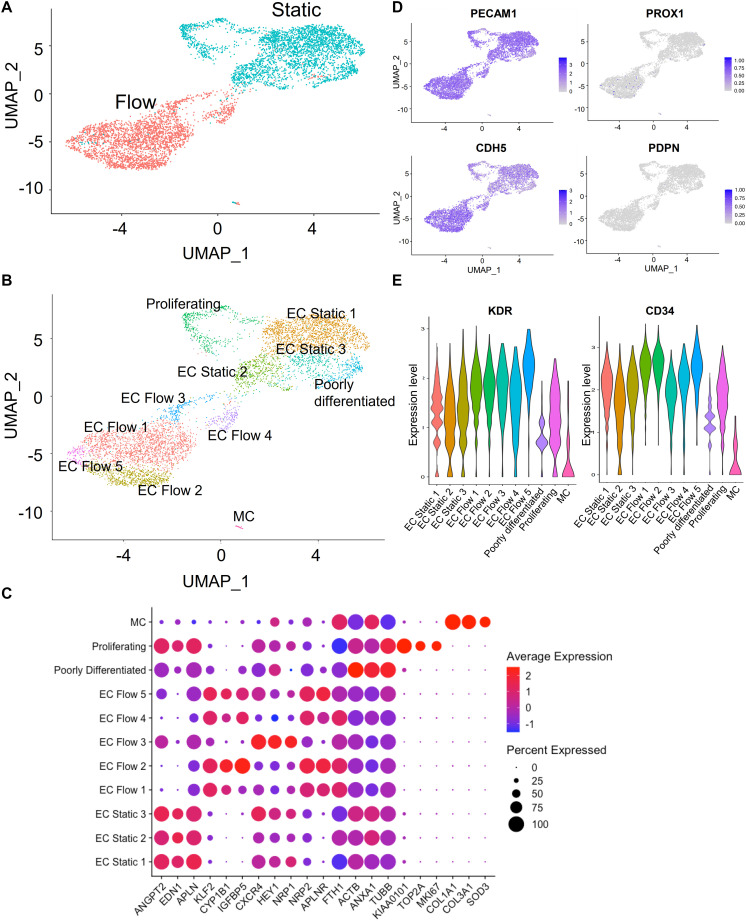
Characterization of single-cell RNA sequencing clusters (HEL47.2 cells). **(A)**, UMAP (Uniform Manifold Approximation and Projection) plot of flow and static cells. **(B)**, UMAP plot of the delineated clusters. **(C)**, Dot plot for marker genes of the delineated clusters. **(D)**, Feature plot of blood EC (*PECAM1, CDH5*) and lymphatic EC (*PROX1, PDPN*) EC marker gene expression. **(E)**, Violin plot of EC marker genes (*KDR, CD34*) in all identified clusters.

A total of 11 clusters were identified in the aggregated data of both flow and static conditions (Figures 2A–C). Most hiPS-ECs had high expression of the endothelial cell markers *CD34*, *PECAM1*, *KDR*, and *CDH5* (Figures 2D,E). The expression of lymphatic EC genes (*PROX1* and *PDPN*) was very low or absent, demonstrating that the protocol used in our study does not produce lymphatic ECs (Figure 2D). Static hiPS-ECs formed five distinct clusters, of which one consisted of proliferating cells with high expression of the cell cycle genes *MKI67*, *TOP2A*, and *BIRC5* (Proliferating) and one with markedly lower EC gene expression, which we named as Poorly differentiated (Figures 2B,C). The proliferating cell cluster contained almost exclusively cells from the static culture, reflecting the flow-induced inhibition of EC proliferation. The other three clusters were labeled as Static EC 1–3. Five distinct flow clusters were identified (EC Flow 1–5). Finally, a very small cluster containing few cells from both conditions expressed a mesenchymal cell phenotype with high expression of *TAGLN*, and *ACTA2* (MC, Mesenchymal cells) (Figure 2B). Clustering of the HEL24.3 hiPS-ECs is presented in the Supplementary Figure S1.

### Single-Cell and Bulk RNA Sequencing Reveal Similar Responses in hiPS-ECs Compared to Primary ECs

Flow induced upregulation of 171 genes, and downregulation of 136 genes in the scRNASeq analysis (genes significantly changed in both cell lines, Supplementary Table S1A). The flow-experiment was replicated with four hiPS-EC-lines (HEL47.2, HEL24.3, HEL46.11, and K1), and analyzed with bulk RNASeq with deeper sequencing depth, allowing detection of low-abundant genes. In the bulk RNASeq analysis, 656 significantly upregulated and 525 downregulated genes in response to flow were identified (Supplementary Table S1B). The most highly induced genes in both analyses included several known flow-responsive genes such as *KLF2* and *CYP1B1*, demonstrating that the hiPS-ECs responded to flow in a similar manner as primary ECs (Figure 2C and Supplementary Tables S1A–C). When the scRNASeq and bulk RNASeq data were analyzed together, in total 99 genes were upregulated and 54 genes were downregulated in all data sets, forming a core of the flow-induced genes in hiPS-ECs (Supplementary Table S1C).

### Single-Cell RNA-Sequencing of Flow-Exposed Cells Identifies Arterial- and Venous-Like hiPS-ECs

**FIGURE 3:**
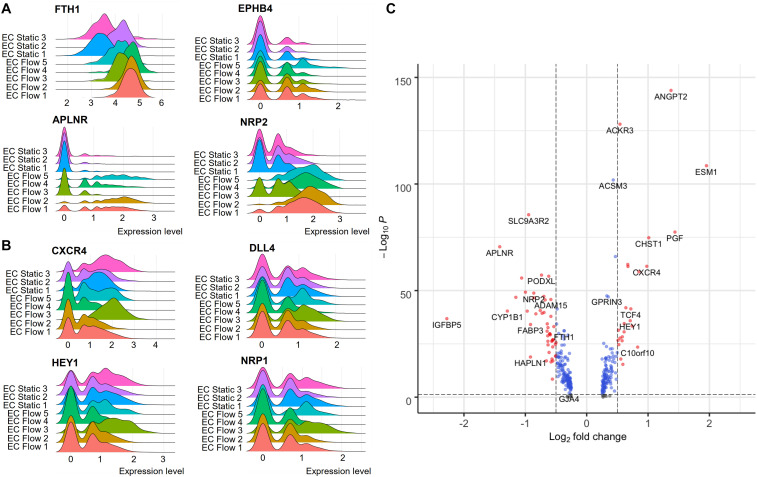
The expression of venous and arterial genes in hiPSC-ECs flow and static clusters (HEL47.2). **(A)**, Venous genes. **(B)**, Arterial genes. **(C)**, Volcano plot on the differential expression of flow arterial-like cluster 3 genes compared to all the other flow clusters.

According to the scRNASeq analysis, flow increased the expression of characteristic EC markers (Figures 2D,E), and both arterial and venous EC markers. HEL47.2 EC Flow clusters 1–2 and 4–5 had higher expression of venous genes (Figure 3A), while the cluster EC Flow 3 had a higher expression of arterial genes (Figure 3B). Similar clustering was found in the HEL24.3 hiPS-ECs with one arterial-like cluster (Supplementary Figure S2). In the bulk RNASeq data, where the effects of flow were studied as a single population, significant upregulation was found in the venous markers *NRP2*, *FTH1*, and *EPHB4*, and the arterial marker *NOTCH1* (Supplementary Table S1B). Except for the scRNASeq clusters EC Flow 3 in the HEL47.2 cell line and EC Flow 5 in the HEL24.3 cell line, which showed more arterial phenotype, the expression of other arterial genes than *NOTCH1* were not significantly changed in the flow hiPS-ECs compared to static hiPS-ECs. Interestingly, these arterial-like clusters showed smaller responses to flow than the other flow clusters based on the expression of the flow-induced genes *KLF2*, *CYP1B1*, and *IGFBP5* (Figure 2C). However, these cells still clearly differed from the static cells (Figure 2C). Compared to the other flow clusters, these cells had increased expression of arterial (*ACKR3, CXCR4, HEY1, GJA1*, and *HES1*) and EC activation markers (*ANGPT2, ESM1*, and *PGF*) (Figure 3C). These findings demonstrate the power of scRNASeq, as the arterial-like cluster was relatively small, and these differences cannot be detected from the bulk RNASeq data.

### Flow-Exposed hiPS-ECs Represent a More Stable and Homeostatic EC Phenotype

**FIGURE 4:**
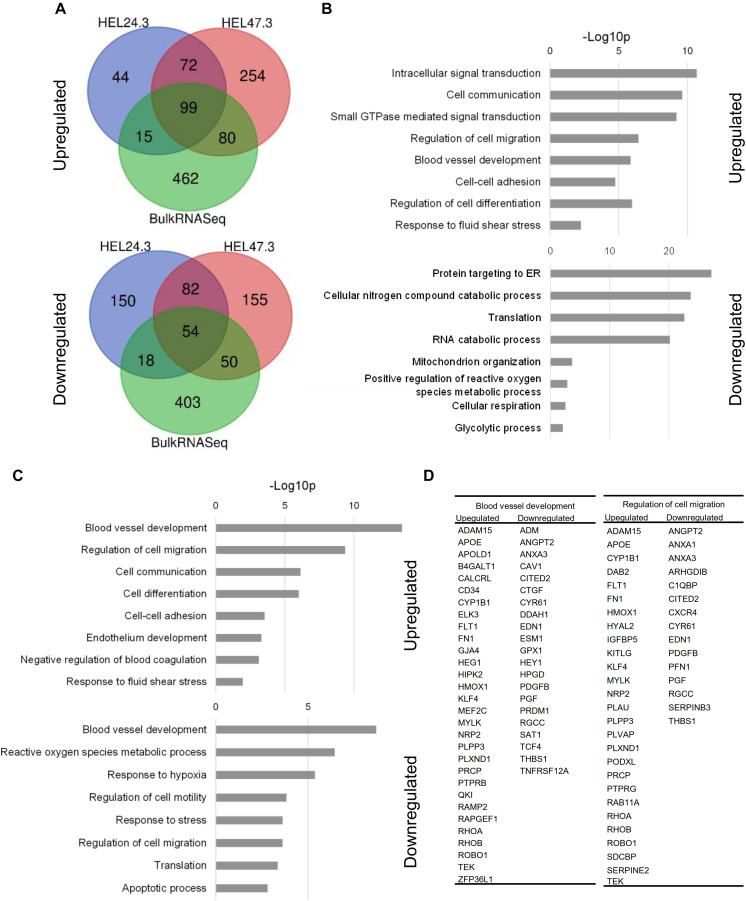
Gene Ontology (GO) analysis of the scRNASeq and bulk RNASeq data (DAVID Bioinformatics Resources). **(A)**, Venn diagram of upregulated and downregulated genes shared between scRNASeq and bulk RNASeq. **(B)**, Upregulated and downregulated GO terms in bulk RNASeq data **(C)**, Upregulated and downregulated GO terms in scRNASeq data. **(D)**, A list of genes included in the GO term categories that were both upregulated and downregulated.

Shear stress activated pathways associated with blood vessel development, cell migration, cell communication, cell-cell adhesion, and fluid shear stress responses, and these replicated in both scRNASeq (HEL47.2 and HEL24.3 combined) and bulk RNASeq analyses (Figures 4A–C). The repressed gene ontologies (GO) in scRNASeq included angiogenesis, reactive oxygen species metabolism, hypoxia response, cell cycle regulation and response to stress. In the bulk RNASeq, in addition to these, pathways related to protein translation and RNA catabolism were significantly downregulated (Figure 4B). In the scRNASeq GO analysis, two categories came up among both upregulated and downregulated genes (blood vessel development and regulation of cell migration) with different affected genes (Figure 4D). Upregulated genes represented flow-response and stabilization of the vasculature and downregulated genes were related to EC activation and proliferation.

**FIGURE 5:**
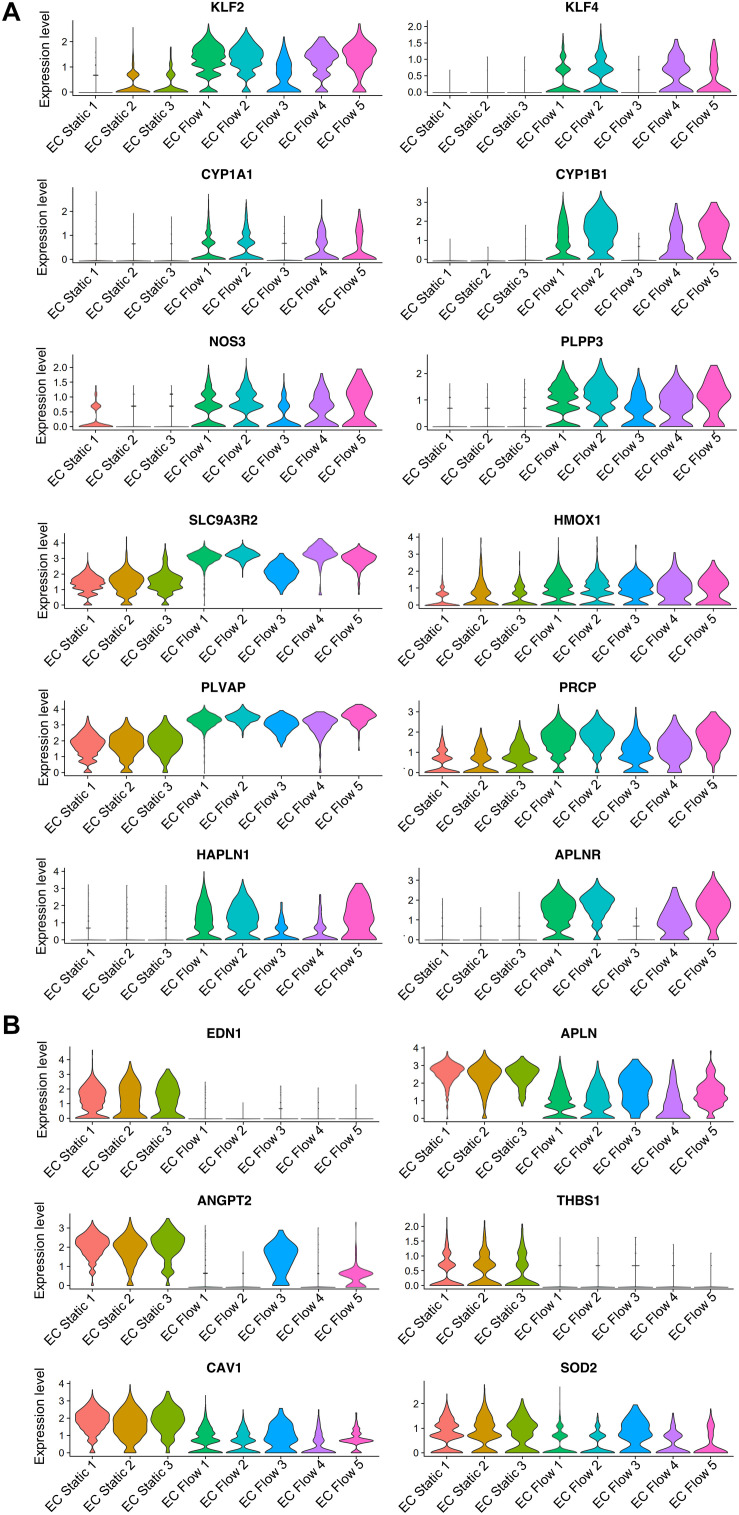
The effect of flow on hiPS-EC gene expression (HEL47.2). **(A)**, Violin plots for selected genes upregulated by flow. **(B)**, Downregulated genes in response to flow.

The expression of known flow-induced genes *KLF2*, *KLF4*, and *eNOS* (*NOS3*), which regulate vascular tone (Sangwung et al., 2017), were highly upregulated in flow (Figure 5A and Supplementary Tables S1A–C). In addition, the expression of anti-atherogenic genes (*CYP1A1*, *CYP1B1*, and *PLPP3*) (Conway et al., 2009; Mueller et al., 2019), genes that promote vascular homeostasis and EC survival (*SLC9A3R2, PODXL*, and *ADAM15*) (Bhattacharya et al., 2012; Horrillo et al., 2016; Babendreyer et al., 2019) and stress response markers (*HMOX1* and *NQO1*) (Dinkova-Kostova and Talalay, 2000; Issan et al., 2014) were upregulated by flow (Figure 5A and Supplementary Tables S1A–C). In contrast, shear stress significantly downregulated the vascular tone regulator *EDN1* (Yanagisawa et al., 1988) in all flow clusters (Figure 5B and Supplementary Tables S1A–C).

**FIGURE 6:**
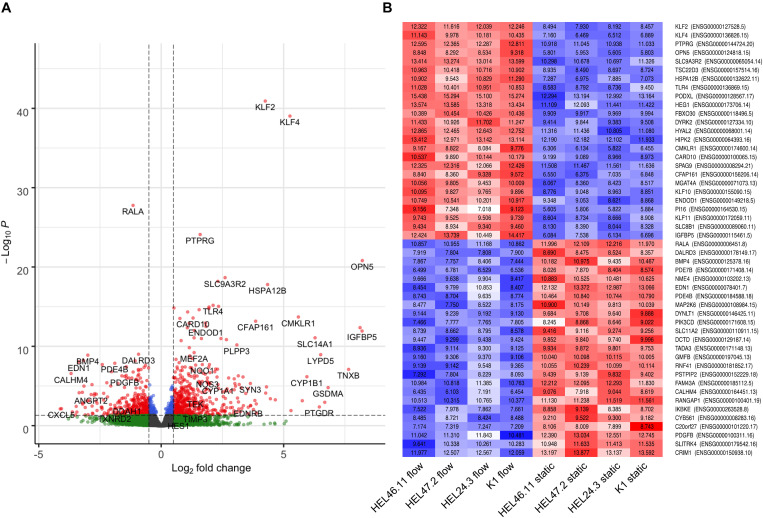
Bulk RNASeq analysis of the effect of flow compared to static condition. **(A)**, A volcano plot of differentially expressed genes in flow compared to static hiPS-ECs. **(B)**, Heat map of the 50 most significantly changed genes by flow. Red denotes upregulation and blue downregulation.

Flow promoted quiescence in hiPS-ECs, demonstrated by downregulation of several angiogenesis and EC activation marker genes (*APLN, ANGPT2*, *CITED2, DDAH1* and *THBS1*) (Freedman et al., 2003; Smadja et al., 2011; Trittmann et al., 2019; Masoud et al., 2020) (Figure 5B and Supplementary Tables S1A–C) and by the upregulation of *TEK* (*TIE2*), that contributes to the maintenance of vascular quiescence (Augustin et al., 2009) (Supplementary Tables S1A–C). Vascular endothelial protein tyrosine phosphatase (*VE-PTP*, also known as *PTPRB*), that regulates blood vessel remodeling and angiogenesis (Küppers et al., 2014), was also markedly induced by flow together with other PTPs *PTPRG* and *PTPRE* (Supplementary Tables S1A–C and Figure 6A).

Also, several other genes that are implicated in vascular health and regeneration (*PRCP*, *APLNR*, *PLVAP*, and *HAPLN1*) (Wirrig et al., 2007; Adams et al., 2013; Deshwar et al., 2016; Guo et al., 2016) were induced by flow (Figure 5A and Supplementary Tables S1A–C). Moreover, shear stress induced downregulation of atherogenic *CAV1* (Melchionna et al., 2005; Fernández-Hernando et al., 2010) and *SOD2* (Ohashi et al., 2006) (Figure 5B and Supplementary Table S1A). A small GTPase *RALA*, which has previously been shown to be repressed by *KLF2* (Dekker et al., 2005), was the most downregulated gene by flow in the bulk RNASeq (Figure 6A and Supplementary Table S1B). In addition, *BMP4* (Helbing et al., 2017) and *PDGFB* (Tisato et al., 2013), which are related to inflammatory responses in ECs, were among the most repressed genes by flow (Figure 6B and Supplementary Table S1B).

Flow also affected metabolic genes in hiPS-ECs, as it increased the expression of glucose transporters *GLUT1* (*SLC2A1*) and *GLUT3* (*SLC2A3*) as well as fatty acid handling genes *FABP3* and *PLIN2* (Supplementary Tables S1A–C).

The respective results for the HEL24.3 cell line are presented in the Supplementary Figure S3.

### Flow Induces NOTCH-Signaling Especially in Arterial-Like hiPS-ECs

**FIGURE 7:**
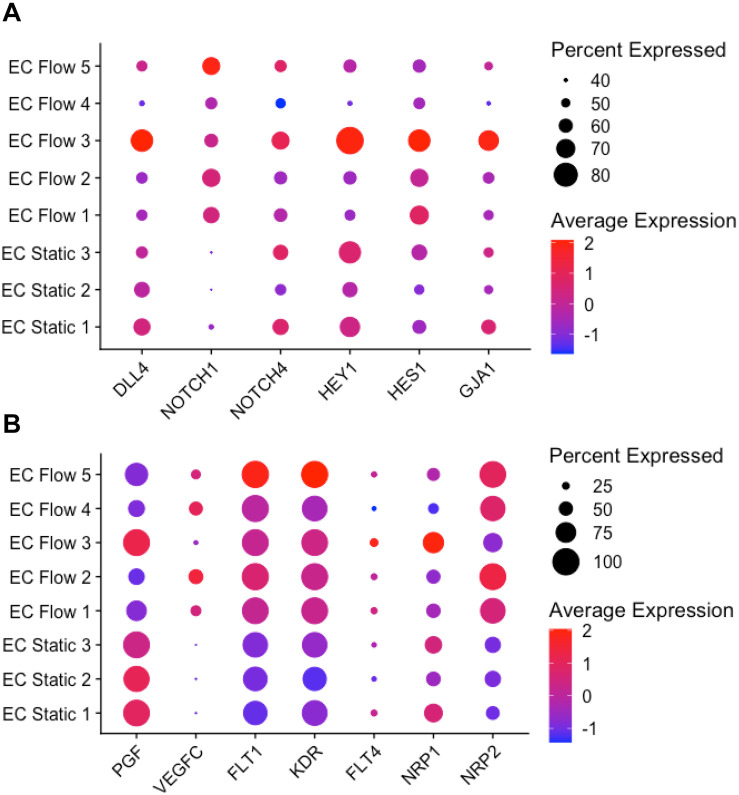
NOTCH and VEGF pathway gene expressions in scRNASeq (HEL47.2). **(A)**, NOTCH pathway gene expression in flow and static clusters. **(B)**, VEGF pathway genes in flow and static clusters.

Shear stress is a known NOTCH-pathway activator inducing increased *NOTCH1* expression. NOTCH1 has recently been identified as an important mechanosensor in ECs (Mack et al., 2017; Polacheck et al., 2017). Compared to static hiPS-ECs, altered expression of several NOTCH pathway genes were observed in the hiPS-ECs exposed to flow. Interestingly, the effects were different in the venous-like (EC Flow 1–2, 4–5) and arterial-like (EC Flow 3) clusters. The expression levels of *NOTCH1* were upregulated in all flow hiPS-EC clusters, but *NOTCH4* only in the arterial-like cluster. The notch-ligand *DLL4* was upregulated in the arterial-like hiPS-ECs in flow, whereas the expression level was repressed in the venous-like hiPS-ECs compared to hiPS-ECs in static conditions (Figure 7A). Likewise, the expression of the NOTCH-targets *HEY1*, *HES1*, and *GJA1* was induced in the arterial-like Flow EC 3. The respective results for HEL24.3 cells are presented in Supplementary Figure S4.

### Flow Altered the Expression of VEGF Signaling Pathway Genes

Markedly reduced expression of the VEGF pathway ligand *PGF* was observed in flow compared to static hiPS-ECs (Figure 7B), whereas *VEGFC* was upregulated by flow, except in the Flow EC 3 cluster. The VEGF receptors *FLT1* (*VEGFR1*), *KDR* (*VEGFR2*), and *FLT4* (*VEGFR3*) tended to be induced by flow in most clusters (Figure 7B). The VEGF co-receptor *NRP2* was upregulated in all flow clusters, whereas *NRP1* was increased specifically in Flow EC 3 cluster (the arterial type cluster). The respective results for HEL24.3 cells are presented in Supplementary Figure S4.

## Discussion

Patient derived hiPS-ECs are increasingly used in disease modeling to study specific disease related phenotypes and genotype-phenotype correlations. However, hiPS-ECs do not gain full maturity and identity compared to ECs *in vivo*, where ECs are exposed to mechanical forces of blood flow such as shear stress and pulsatile pressure, and paracrine signals from neighboring parenchymal cells. In addition, the efficiency of differentiation as well as maturity of cells vary depending on the protocol used. Here we demonstrate that differentiating hiPSCs to hiPS-ECs results in a population of cells with cobble-stone morphology and high expression of EC molecular markers. In addition, these cells are able to form vascular tube structures in 3D and take up oxidized LDL. We show that the hiPS-ECs have high plasticity as they adapt to laminar flow-induced shear stress by entering quiescence and presenting with a more homogenous and homeostatically stable phenotype. Interestingly, the expression of both arterial and venous genes increased in flow, and single-cell analysis and clustering revealed flow-exposed subpopulations with transcriptomic profiles toward either a more venous or arterial identity. Our results highlight the importance of single-cell RNA sequencing to distinguish different phenotypes of hiPSC-derived ECs.

Based on their gene expression profiles, hiPS-ECs are more similar to embryonic ECs when compared to primary ECs (Rufaihah et al., 2013; Orlova et al., 2014a; Vazão et al., 2017). The expression levels of the endothelial progenitor marker *CD34* were high in our hiPS-ECs, demonstrating an immature nature of these cells. This feature potentially results in plasticity to further develop to, or even transfer between arterial and venous phenotypes according to various external cues (Rufaihah et al., 2013; Ikuno et al., 2017). This is an advantage when modeling the development and maturation of ECs and vasculature. In addition, due to these properties, hiPS-ECs can potentially provide a relevant model for screening for embryonic vascular toxicity (Vazão et al., 2017), to model diseases associated with immature vasculature, or to study the effect of EC responses to abnormal flow conditions during development.

hiPS-ECs cultured in static conditions expressed more arterial than venous markers, which is consistent with previous reports (Paik et al., 2018; Vilà-González et al., 2019). However, the cells did not cluster according to arterial or venous phenotype in static culture. Single-cell sequencing of flow-exposed hiPS-ECs revealed increased expression of several arterial and venous genes, with higher induction seen in venous markers, which is in accordance with previous studies of hiPS-ECs (Ohtani-Kaneko et al., 2017). The expression pattern was replicated in the bulk RNASeq data for the venous markers *NRP2, FTH1*, and *EPHB4*, and the arterial marker *NOTCH1*. Importantly, single-cell RNA-sequencing revealed several hiPS-EC-subclusters in the flow exposed cells, of which one was clearly arterial-like and the others venous-like, underlining the responsiveness of the cells to external stimuli and also the heterogeneity of the phenotypic change. When compared to primary ECs *in vivo*, hiPS-ECs in culture do not show organotypic features or clear separation of arterial, venous or capillary ECs (Kalucka et al., 2020), thus the clusters are not directly comparable to *in vivo* primary EC clusters. The expression levels of the lymphatic EC markers *PROX1* and *PDPN* were very low, indicating that there were no lymphatic ECs among the hiPS-ECs. A small number of cells expressed the smooth muscle cell marker *ACTA2*, and these cells clustered far from other hiPS-ECs and were discarded in further analyses.

As expected, flow activated known vascular tone regulators, such as *KLF2* (Dekker et al., 2002) and *KLF4* (Sangwung et al., 2017), and shear responsive anti-atherogenic genes, such as *CYP1A1*, *CYP1B1*, and *PLPP3* (Conway et al., 2009; Mueller et al., 2019). This demonstrates that the hiPS-ECs are plastic and can adapt to flow-induced changes similarly to primary ECs. Interestingly, the cells, which had the lowest response to flow. e.g., attenuated upregulation of *KLF2* and *CYP1B1*, acquired more of an arterial-like phenotype. In primary ECs and *in vivo*, KLF2 has been shown as a positive transcriptional regulator of shear-dependent endothelial function for example through upregulation of endothelial nitric oxide synthase (*eNOS*), and downregulation of the vasoconstrictor *EDN1* and small GTPase *RALA* (Bhattacharya et al., 2005; Dekker et al., 2005). These effects were also observed in the present study.

Other changes indicating a stabilizing effect of flow were also seen, such as the downregulation of *SOD2*, *PGF*, and *CAV1*. SOD2 protects against oxidative stress and endothelial dysfunction (Ohashi et al., 2006). Hypoxia increases PGF expression in human myocardium (Torry et al., 2009), and pathological cardiac conditions such as ischemic cardiomyopathy or acute myocardial infarction result in elevated plasma levels of PGF (Iwama et al., 2006; Nakamura et al., 2006). Overexpression of the structural protein CAV-1 in mouse ECs lead to progression of atherosclerosis, while the absence of it reduces the progression of atherosclerosis (Fernández-Hernando et al., 2010). Three protein tyrosine phosphatases (*PTPRB*, *PTPRG*, and *PTPRE*) were strongly induced by flow. PTPRB (VE-PTP) activity enhances VE-cadherin-mediated adhesion and promotes endothelial barrier function (Nawroth et al., 2002), and it is also an important regulator of TEK, VEGFR2 and PECAM1 activity (Küppers et al., 2014), indicating EC stabilization. We also identified *SLC9A3R2 (NHERF2*) as a novel flow-response gene by both single-cell and bulk RNASeq. It has previously been shown to be expressed specifically in ECs (Wallgard et al., 2008) and to regulate vascular homeostasis (Bhattacharya et al., 2012; Schrimpf et al., 2012). Interestingly, in kidney MDCK cells, NHERF2 was shown to bind to podocalyxin (PODXL), another gene significantly induced by flow in our model, and to regulate epithelial cell polarization (Meder et al., 2005). It is likely that these genes act together also in ECs mediating the flow-induced cellular polarization. Thus, our findings show that many genes necessary for stability, barrier function, polarization and regeneration are highly responsive to flow in hiPS-ECs.

Laminar shear stress -induced KLF2 activation has been shown to modulate EC metabolism by reducing glucose uptake and glycolysis, which contributes to EC quiescence (Doddaballapur et al., 2015). Pathway analysis of the bulk RNASeq data revealed that flow repressed glycolysis-related genes also in hiPS-ECs. However, we observed increased expression of glucose transporters *GLUT1* and *GLUT3* as well as fatty acid handling genes *FATP3* and *PLIN2* in flow-exposed hiPS-ECs in all three experiments (Supplementary Tables S1A–C). As glucose uptake is mainly regulated by translocation of GLUTs to cell membrane, and not by transcription, we think that the upregulation of glucose and fatty acid transport/storage genes mainly reflect the maturation of flow-exposed hiPS-ECs compared to the static cells, and not regulation of glucose uptake or glycolysis.

VEGF and NOTCH signaling are both essential for blood and lymphatic vasculature growth and specification. Our data showed that the VEGF receptors *FLT1* and *KDR* were highly expressed in all hiPS-ECs. Flow induced significant repression of *PGF* and a slight upregulation of *VEGFC*. In addition, the co-receptor *NRP2* was markedly induced in the venous-like clusters, whereas *NRP1* was upregulated specifically in the arterial-like cluster. Furthermore, markers of EC activation and proliferation were significantly repressed by flow. These changes indicate a more stable and quiescent EC phenotype. *NOTCH1*, which has been shown to act as a mechanosensor in ECs (Mack et al., 2017), was highly upregulated by shear stress in all flow clusters in hiPS-ECs. The NOTCH pathway ligands and effectors *DLL4*, *HEY1*, *HES1*, and *GJA1* were induced only in the arterial-like Flow EC 3 cluster, but were found to be expressed in all clusters to some level. Recently, NOTCH activation was found to be important for cell cycle arrest and arterial specification in ECs (Fang et al., 2017).

Analysis at the single-cell level shows that as a group, the flow-stimulated ECs are more homogenous as compared to hiPS-ECs grown in static conditions. The clusters that were identified as poorly differentiated were absent in the flow-exposed cells. It is possible that flow results in further improved differentiation of these cells, or that subpopulations of the iPS-ECs in static conditions have transdifferentiation properties, which are suppressed by flow. This is an important finding considering e.g., disease modeling, as the comparison between healthy and diseased cells would have much less noise in the data when studied under flow conditions. This also mimics better the *in vivo* environment, as ECs are constantly exposed to shear stress.

This study is limited by the number of iPS-cell lines used, mainly due to the high costs of single-cell RNA-sequencing. However, the obtained results were highly reproducible in scRNASeq analysis of both cell lines as well as in a replication experiment with bulk RNAseq of 4 different hiPS cell lines. The disadvantages of using iPSC-derived ECs compared to primary ECs or cell lines is the immature nature of the cells, their tendency for transdifferentiation, the low proliferation capacity over passaging and the costs to produce them. However, in disease modeling they are superior to study the interactions between cell types, as all cell types can be derived from the same patient carrying the same genetic variants. This is why the development of better iPS-EC models are highly needed. Our study demonstrates the importance of flow for the stability and homeostasis of hiPS-ECs.

In summary, our study showed that exposing hiPS-ECs to laminar shear stress promotes a more stable and quiescent EC phenotype, which was more homogenous than hiPS-ECs grown under static conditions. The immature nature of hiPS-ECs is an advantage for modeling the effects of flow on EC phenotype and maturation. Our results also demonstrate that the flow responses are highly consistent in different healthy hiPS-EC lines, similarly to what was previously shown for barrier function and inflammatory responses (Halaidych et al., 2018).

## Data Availability Statement

The datasets presented in this study can be found in online repositories. The names of the repository/repositories and accession number(s) can be found below: https://www.ncbi.nlm.nih.gov/geo/, GSE150741 https://www.ncbi.nlm.nih.gov/geo/, GSE150740.

## Ethics Statement

The studies involving human participants were reviewed and approved by the Ethics Committee of Helsinki and Uusimaa Hospital District. The patients/participants provided their written informed consent to participate in this study.

## Author Contributions

EH and RK contributed to the conceptualization, funding acquisition, and supervision. EH contributed to the data curation, formal analysis, project administration, and software. MA, EH, and LA contributed to the investigation. EH and MA contributed to the methodology and visualization. EH and RK contributed to the resources. EH, RK, and MA contributed to the writing of the original draft. EH, MA, and RK contributed to the writing – review and editing. All authors contributed to the article and approved the submitted version.

## Conflict of Interest

The authors declare that the research was conducted in the absence of any commercial or financial relationships that could be construed as a potential conflict of interest.

## Supplementary Material

Supplementary Figure 1 Characterization of single-cell RNA sequencing clusters in HEL24.3 cells. **(A)** UMAP (Uniform Manifold Approximation and Projection) plot of flow and static cells. **(B)** UMAP plot of the delineated clusters. **(C)** Dot plot of the marker genes in delineated clusters. **(D)** Feature plot of blood (*PECAM1, CDH5*) and lymphatic (*PROX1, PDPN*) EC marker gene expression. **(E)** Violin plot of EC marker genes in all identified clusters.

Supplementary Figure 2 The expression of venous and arterial genes in hiPSC-EC flow and static clusters (HEL24.3). **(A)** Venous genes. **(B)** Arterial genes.

Supplementary Figure 3 The effect of flow on hiPSC-EC gene expression (HEL24.3). **(A)** Selected genes upregulated by the exposure to flow. **(B)** Genes downregulated in response to flow.

Supplementary Figure 4 NOTCH and VEGF pathway gene expression in scRNASeq (HEL24.3). **(A)** NOTCH pathway genes expressed in flow and static clusters. **(B)** VEGF pathway genes expressed in flow and static clusters.

Supplementary Table 1 List of differentially expressed genes upon flow exposure in **(A)** bulk RNASeq data, **(B)** scRNASeq data and **(C)** in combined analysis of bulk and single-cell RNASeq data.
