# Supplementary material for: Flow-Induced Transcriptomic Remodeling of Endothelial Cells Derived From Human Induced Pluripotent Stem Cells
Source: Front Physiol. 2020 Oct 15;11:591450. doi: 10.3389/fphys.2020.591450 (PMC7593792; doi:10.3389/fphys.2020.591450)
Supplement: Supplementary Figure 1 — Characterization of single-cell RNA sequencing clusters in HEL24.3 cells. (A) UMAP (Uniform Manifold Approximation and Projection) plot of flow and static cells. (B) UMAP plot of the delineated clusters. (C) Dot plot of the marker genes in delineated clusters. (D) Feature plot of blood (PECAM1, CDH5) and lymphatic (PROX1, PDPN) EC marker gene expression. (E) Violin plot of EC marker genes in all identified clusters. [file Data_Sheet_1.PDF]

Figure S1

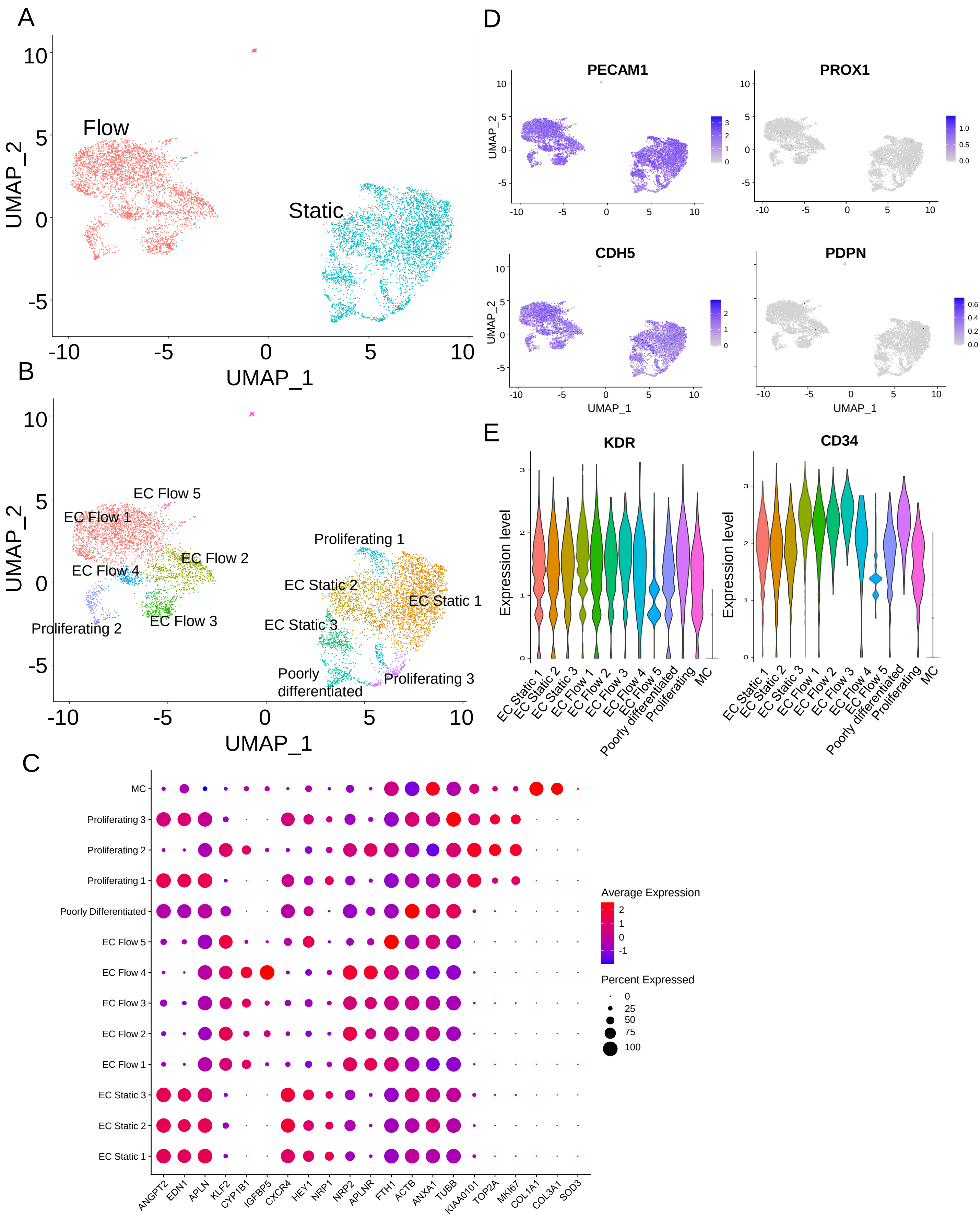

Figure S1. Characterization of single-cell RNA sequencing clusters in HEL24.3 cells. (**A**), UMAP (Uniform Manifold Approximation and Projection) plot of flow and static cells. (**B**), UMAP plot of the delineated clusters. (**C**), Dot plot of the marker genes in delineated clusters. (**D**), Feature plot of blood (*PECAM1*, *CDH5*) and lymphatic (*PROX1*, *PDPN*) EC marker gene expression. (E), Violin plot of EC marker genes in all identified clusters.

Figure S2

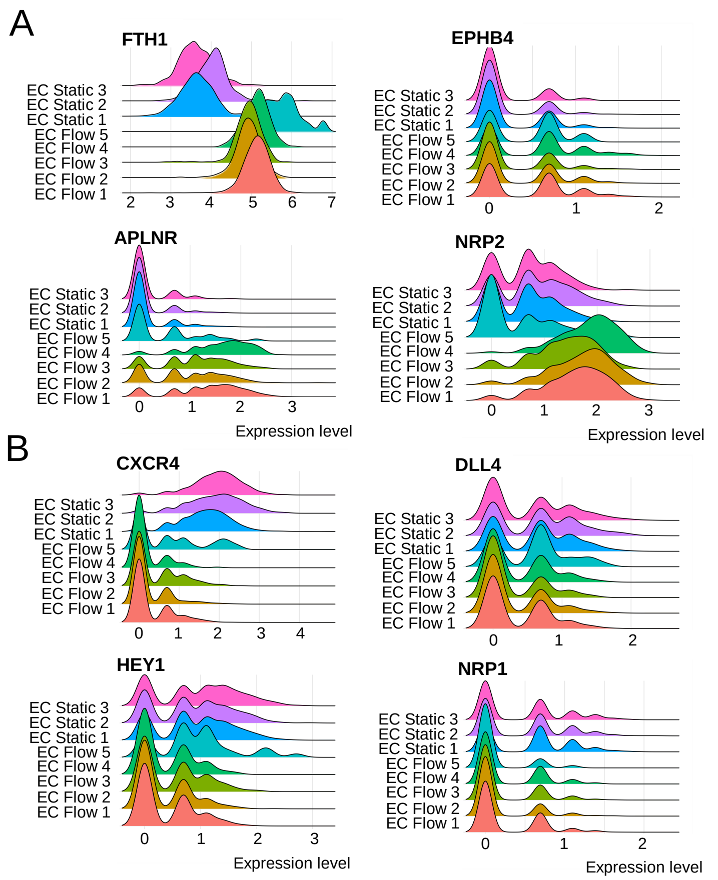

Figure S2. The expression of venous and arterial genes in hiPSC-EC flow and static clusters (HEL24.3). (**A**), Venous genes. (**B**), Arterial genes.

Figure S3

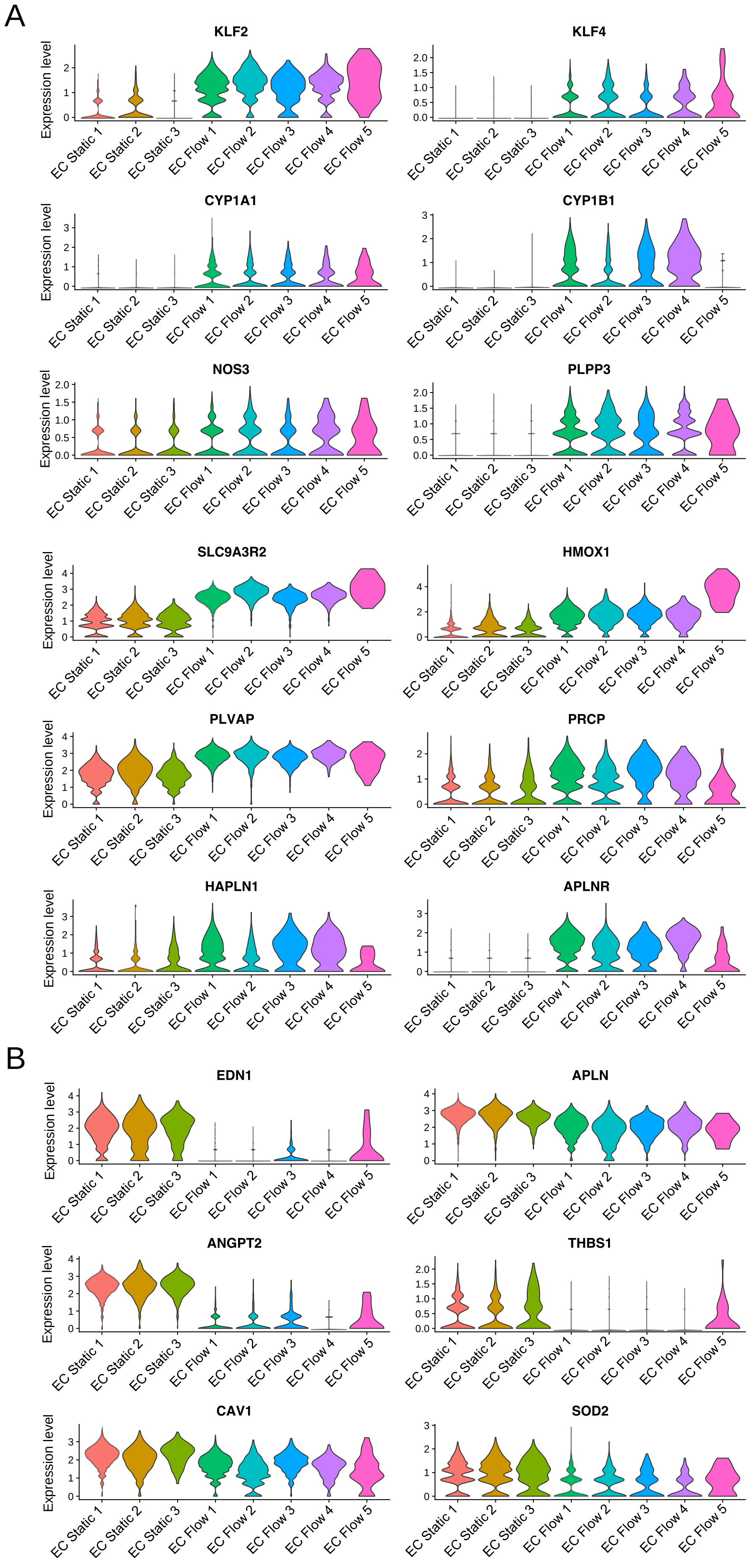

Figure S3. The effect of flow on hiPSC-EC gene expression (HEL24.3). (**A**), Selected genes upregulated by the exposure to flow. (**B**), Genes downregulated in response to flow.

Figure S4

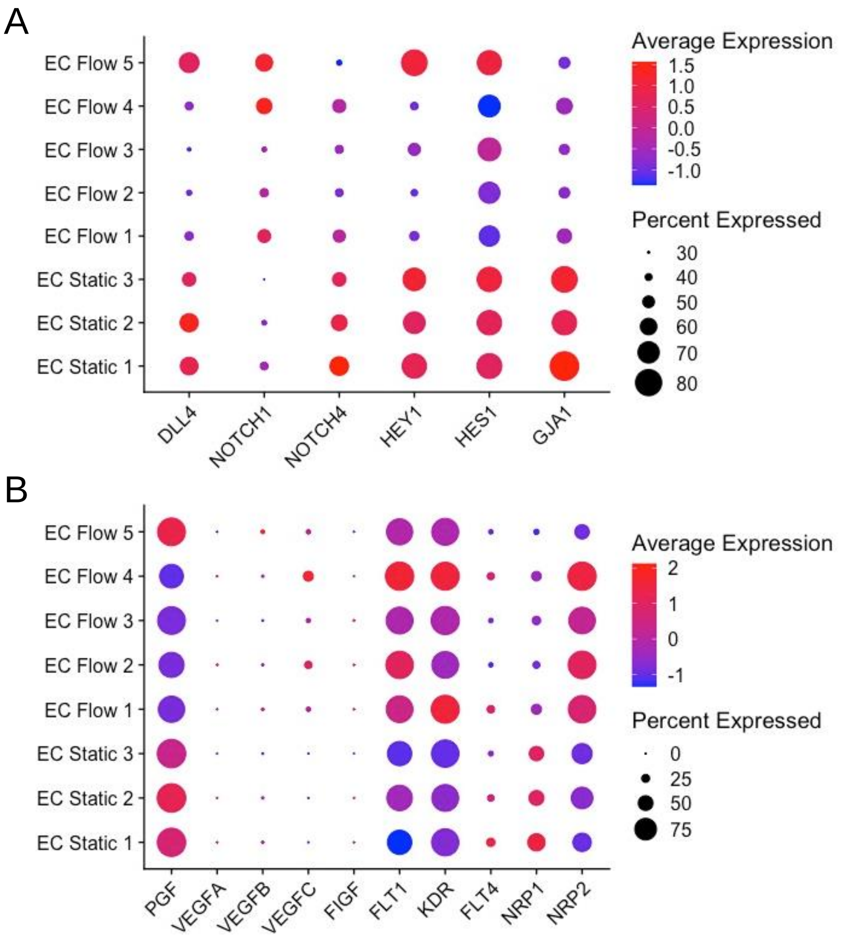

Figure S4. NOTCH and VEGF pathway gene expression in scRNASeq (HEL24.3). (**A**), NOTCH pathway genes expressed in flow and static clusters. (**B**), VEGF pathway genes expressed in flow and static clusters.
